# Dysregulation of lncRNA MEG3/miR-21-5p Axis Impairs SOX5 Expression in Osteoarthritis

**DOI:** 10.3390/genes17070748

**Published:** 2026-06-29

**Authors:** Stavroula Kyriakaki, Charalampos Balis, Aliki-Alexandra Papageorgiou, Vasileios Konteles, Nikolaos Stefanou, Sokratis E. Varitimidis, Aspasia Tsezou, Ioanna Papathanasiou

**Affiliations:** 1 Laboratory of Cytogenetics and Molecular Genetics, Faculty of Medicine, School of Health Sciences, University of Thessaly, Biopolis, 41500 Larissa, Greece; skyriakaki@uth.gr (S.K.); chbalis@uth.gr (C.B.); alipapageorgiou@uth.gr (A.-A.P.); kontelesb@gmail.com (V.K.); atsezou@uth.gr (A.T.); 2 Department of Orthopaedic Surgery, Faculty of Medicine, School of Health Sciences, University of Thessaly, Biopolis, 41500 Larissa, Greece; nikvsteph@gmail.com (N.S.); svaritimidis@gmail.com (S.E.V.)

**Keywords:** LncRNAs, MEG3, miR-21-5p, SOX5, osteoarthritis

## Abstract

Background/Objectives: Emerging evidence shows long non-coding RNAs (lncRNAs) as critical regulators of osteoarthritis (OA) progression, often acting in complex networks with microRNAs (miRNAs). In our study, we investigated the potential regulatory function of the lncRNA MEG3/miR-21-5p axis in the OA phenotype of chondrocytes. Methods: Differential gene expression analysis in damaged vs. intact cartilage was performed, re-analyzing existing public RNA-seq data. MiRTarBase, LncRNADisease, and Open Targets databases were utilized to identify miR-21-5p target genes and OA-associated lncRNAs and genes. Functional enrichment analysis and protein–protein interaction (PPI) network construction were performed using the DAVID and STRING databases, respectively. MEG3, miR-21-5p, SOX5, COL2A1 and ACAN mRNA expressions were assessed by qRT-PCR. The role of the MEG3/miR-21-5p axis in OA chondrocytes was examined using transfection experiments. Results: Eighty-one lncRNAs displayed significant differences in expression between damaged and intact cartilage, including MEG3. Bioinformatic analysis indicated that MEG3 interacts with miR-21-5p, while SOX5 was identified to be a putative target of miR-21-5p. MEG3 and SOX5 expression levels were significantly downregulated in OA chondrocytes, whereas miR-21-5p expression was upregulated. Silencing of MEG3 resulted in increased miR-21-5p levels in chondrocytes. Conversely, inhibition of miR-21-5p led to increased SOX5 expression and anabolic markers COL2A1 and ACAN. Notably, MEG3 silencing significantly reduced SOX5 expression, an effect that was reversed upon miR-21-5p inhibition. Conclusions: Our findings highlight a potential regulatory role of the dysregulated MEG3/miR-21-5p axis in modulating the anabolic phenotype of chondrocytes through regulation of SOX5 expression. This novel lncRNA/miRNA/mRNA regulatory network may represent a candidate therapeutic axis for knee osteoarthritis.

## 1. Introduction

Osteoarthritis (OA) constitutes a widespread health condition that ranks among the leading causes of disability and chronic pain [1,2]. It is estimated to affect nearly 600 million individuals globally, and this trend is expected to continue upward, reaching 1 billion by 2050 [1,2]. OA is a complex joint disease and affects weight-bearing joints (knee, hip) as well as non-weight-bearing joints (hand, finger) [3]. Several factors, including age, clinical obesity, female sex, mechanical stress and genetic predisposition, are associated with an increased risk of OA and contribute to joint structural decline [4]. Although OA is a highly prevalent disease with socioeconomic impact, there is no effective disease-modifying treatment for osteoarthritis, and current strategies focus on pain management, functional improvement and surgical interventions [4].

OA is considered as a whole-joint degenerative disorder, characterized by pathological manifestations in articular cartilage, synovium, subchondral bone, ligaments and fat pad tissues [5,6]. These pathophysiological changes include progressive cartilage degradation, synovial inflammation, remodeling of the subchondral bone and osteophyte formation [7]. These processes are driven by the crosstalk among the joint tissues through a complex interplay of specific mechanisms as well as by chondrocytes’ dysfunction [5,6,7,8]. The extensive changes in the extracellular matrix (ECM) by the loss of chondrocyte ECM maintenance properties compromise joint function and underly the clinical manifestations of pain and stiffness [8,9,10], reinforcing cartilage degeneration as the central process in OA pathogenesis.

Emerging evidence shows that the epigenetic mechanisms, including DNA methylation, histone modification and regulatory RNAs, act as key mediators of disease progression modulating the expression of genes implicated in OA-related processes [11,12]. Regarding regulatory RNAs, long non-coding RNAs (lncRNAs), which are transcripts exceeding 200 nucleotides, regulate gene expression via cis or trans mechanisms affecting chromatin remodeling, mRNA stability and protein function [13]. Moreover, they form competing endogenous RNA (ceRNA) networks, where they function as “RNA sponges” that act as competing molecules that limit miRNA effects on protein-coding mRNA targets [14]. In OA, numerous lncRNAs exhibit abnormal expression and modulate processes related to OA pathogenesis, often by interacting with OA-related miRNAs, including miR-21, miR-140 and miR-146a [13,15,16,17]. Dysregulated lncRNAs, such as HOX transcript antisense RNA (HOTAIR), H19, Maternally Expressed 3 (MEG3), and Metastasis Associated Lung Adenocarcinoma Transcript 1 (MALAT1), can promote disease progression or facilitate cartilage repair through regulation of OA-associated signaling pathways such as Wnt/β-catenin, NF-κB, and MAPK [15,16,17]. Exploring the function of lncRNAs in OA degenerative processes may provide valuable insights for developing novel biomarkers and future targeted therapy.

This study employs a discovery-based bioinformatic approach using publicly available RNA-seq data to identify osteoarthritis-associated lncRNAs, followed by targeted experimental validation in primary human chondrocytes. Specifically, we investigated the role of the selected candidate lncRNA, MEG3, in the OA phenotype of chondrocytes. Furthermore, we examined the potential regulatory interaction between MEG3 and miR-21-5p, as well as its downstream association with SOX5, aiming to elucidate the molecular mechanisms of the MEG3/miR-21-5p/SOX5 axis and its contribution to OA pathogenesis.

## 2. Materials and Methods

### 2.1. Patients and Sample Collection

Articular cartilage specimens with severe OA damage were collected from 16 individuals undergoing knee replacement surgery at the Orthopedics Clinic of the University Hospital of Larissa. Prior to undergoing surgical intervention, patients were categorized based on radiographic imaging using the Kellgren–Lawrence (K/L) grading system. The K/L score for each patient exceeded 2, indicating the presence of radiographic evidence of knee osteoarthritis. Healthy articular cartilage was obtained from 10 individuals who had undergone surgical procedures for knee fracture repair or amputation. Healthy individuals had no history of joint disease and showed no clinical signs of osteoarthritis (OA) on radiographic examination. Cartilage sections obtained during fracture repair or amputation cases were evaluated by 2 orthopedic surgeons through direct visual inspection and were macroscopically graded as grade 0. Histological assessment was not performed for the control cartilage. Chondral plugs were typically taken from the far outer edges of the femoral condyles, the trochlear groove, the intercondylar eminence and intercondylar fossa-peripheral rims, according to surgical approaches chosen on a case-by-case basis. All healthy subjects were classified preoperatively as Kellgren–Lawrence (KL) grade 0 patients. Table 1 displays the demographic data of the study groups. Healthy individuals and OA patients were age and sex matched. Patients with rheumatoid arthritis or other autoimmune diseases, chondrodysplasias, infection-induced or post-traumatic OA were excluded. Ethics approval was granted from the Local Ethics Committee of the University Hospital of Larissa (No 55277, 15/11/2016; No. 52425, 02/12/2025) in compliance with the ethical standards of the 1975 Declaration of Helsinki.

### 2.2. Cell Culture of Chondrocytes

Chondrocytes were obtained from both osteoarthritic (OA) and healthy articular cartilage, as previously reported [18]. Briefly, the cartilage was digested sequentially with pronase (1 mg/mL) for 30 min, followed by treatment with collagenase P (1 mg/mL) (Roche Applied Science, Mannheim, Germany) for 3 h at 37 °C. Then, chondrocytes were cultured independently in DMEM/F-12 (Dulbecco’s Modified Eagle Medium/Ham’s F-12; Thermo Fisher Scientific, Waltham, MA, USA) containing 10% fetal bovine serum, penicillin (100 IU/mL) and streptomycin (100 μg/mL), all by Thermo Fisher Scientific, Waltham, MA, USA. Cultures were maintained at 37 °C in 5% CO_2_, and fresh medium was added every 3–4 days until the desired confluence was reached. Chondrocytes were maintained in culture for 8–10 days and used at passage 1 to avoid culture-induced dedifferentiation.

### 2.3. Cell Transfection

Independent donor-derived chondrocytes (*n* = 3 biological replicates) were plated in six-well plates and maintained in growth medium without antibiotics. On the following day, when the cells reached 60–80% confluence, they were transfected for 48 h with 50 pmol siRNA against MEG3 (si-MEG3) or negative control (NC-siRNA) (Qiagen, Germantown, MD, USA), or with 25 pmol miR-21-5p inhibitor or negative control inhibitor (NC-inhibitor) (Thermo Fisher Scientific, Waltham, MA, USA). Transfection was performed using Opti-MEM Medium and Lipofectamine^TM^ 2000 (Invitrogen, Life Technologies, Paisley, UK) according to the manufacturer’s instructions. For co-transfection, chondrocytes were first transfected with si-MEG3 (50 pmol) for 48 h, after which the medium was removed and 25 pmol of miR-21-5p inhibitor was added for an additional 48 h.

### 2.4. Differential Analysis of lncRNAs

Using the Gene Expression Omnibus (GEO) database, the GSE114007 dataset was downloaded, which includes knee joint cartilage specimens from 20 knee OA patients and 18 healthy controls [19]. The RNA-seq data was retrieved from the GPL11154 (Illumina, HiSeq 2000, Homo sapiens) and GPL18573 (Illumina NextSeq 500 (Homo sapiens)) platforms. Differential expression analysis was performed in R (version 4.4.2) with the ‘DESeq2’ package (version 1.46.0) [20]. A sequencing platform was included in the DESeq2 design formula to account for potential technical variation between the HiSeq 2000 and NextSeq 500 datasets. LncRNA annotation was performed using Ensembl gene annotations retrieved through the Bioconductor packages AnnotationHub and ensembldb. Gene symbols were mapped to Ensembl records, and gene biotype information was extracted from the corresponding EnsDb database. Prior to differential expression analysis, lowly expressed genes were filtered by retaining only genes with at least five read counts in a minimum of two samples. LncRNAs were considered differentially expressed when the adjusted *p*-value (padj) was below 0.05 and the absolute log2 fold change (log2FC) exceeded 0.5. Volcano plots and heatmaps were produced with the ‘ggplot2’ package (version 4.0.1) [21] to present the differential expression results.

### 2.5. RNA Extraction and Quantitative Real-Time PCR (qRT-PCR)

Total RNA was extracted from cultured cells using TRIzol reagent (Thermo Fisher Scientific, Waltham, MA, USA) according to the manufacturer’s instructions, and RNA concentration was determined using the Qubit Fluorometer (Life Technologies, Waltham, MA, USA). First-strand cDNA was generated using M-MLV reverse transcriptase with Random Primers or specific stem-loop RT primers for U6 and miR-21-5p (Thermo Fisher Scientific, Waltham, MA, USA). Quantitative RT-PCR was carried out using Power SYBR Green PCR Master Mix (Thermo Fisher Scientific, Waltham, MA, USA) and gene-specific primers (Table 2) on an ABI 7300 PCR system, followed by analysis with ABI 7300 system SDS software (version 1.4.0). U6 small nuclear RNA (U6 snRNA) and glyceraldehyde 3-phosphate dehydrogenase (GAPDH) served as internal controls for miR-21-5p and lncRNA/mRNA targets (MEG3, SOX5, COL2A1 and ACAN), respectively. Relative expression was calculated using the 2^−ΔΔCt^ approach [22]. All reactions were run in duplicate.

### 2.6. Computational Prediction of miRNA Targets and Functional Enrichment Analysis

The miRTarBase database [23], a large-scale repository of experimentally validated miRNA-target interactions, was used to identify miR-21-5p target genes. To ensure the reliability of the identified targets, only those that had been verified by Western blot, qPCR, and reporter assay were considered. Functional annotation of miRNA target genes was carried out using the Database for Annotation, Visualization, and Integrated Discovery (DAVID), and the genes were categorized according to biological process terms [24]. Enriched pathways associated with miR-21-5p target genes were extracted using the Kyoto Encyclopedia of Genes and Genomes (KEGG) pathway analysis. Enrichment analysis was conducted using the EASE score (a modified Fisher’s exact test) with default parameters in DAVID, and the Homo sapiens genome-wide annotation set was used as the background. Pathways and functions with *p*-value < 0.05 were considered significantly enriched. OA-related genes were retrieved from the platform Open Targets (https://www.opentargets.org/, accessed on 26 January 2026) [25], while OA-associated lncRNAs were obtained from LncRNADisease v3.0 [26]. Venn diagrams were generated using the Interactive Venn tool (https://www.interactivenn.net/, accessed on 1 February 2026) [27].

### 2.7. Protein–Protein Interaction Network

A protein–protein interaction (PPI) network was constructed using miR-21-5p target genes from the STRING database [28], with an interaction score threshold of 0.4. The network was visualized in Cytoscape (version 3.10.3) (https://cytoscape.org/, accessed on 18 February 2026), where nodes denoted genes or proteins and edges represented their interactions. Cluster enrichment analyses were subsequently performed using the ClueGo plugin (version 2.5.10) in Cytoscape.

### 2.8. Statistical Analysis

Statistical analyses were performed using SPSS version 32. Results are reported as mean ± SEM. In box-and-whisker plots, the central line denotes the median, the box corresponds to the interquartile range, and the whiskers indicate the minimum and maximum values. Depending on the data structure, comparisons between groups were analyzed using unpaired or paired Student’s *t*-tests and the Mann–Whitney U-test. Correlations were assessed using Spearman’s rank correlation coefficient. Statistical significance was defined as *p* < 0.05.

## 3. Results

### 3.1. Expression Profile of Long Non-Coding RNAs (lncRNAs) in OA Cartilage

**Figure 1:**
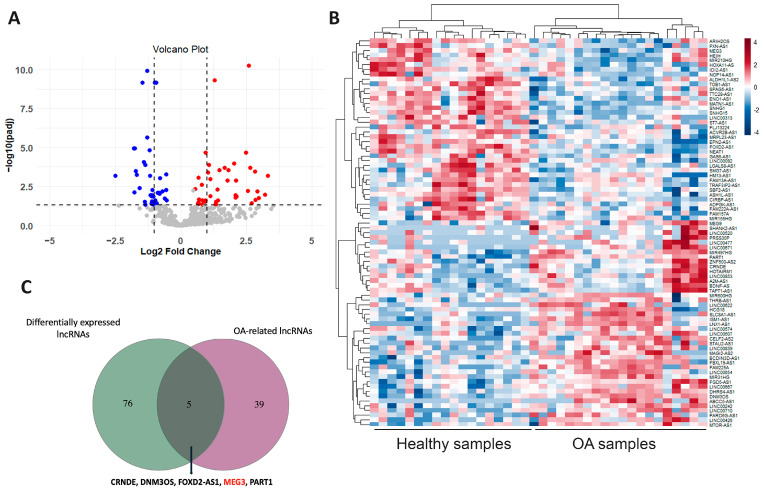
Differentially expressed lncRNAs in OA cartilage. (**A**) Volcano plot illustrating differentially expressed lncRNAs between osteoarthritic (OA) and healthy cartilage (adjusted *p*-value < 0.05; |log2 fold change| > 0.5). (**B**) Heatmap indicating the expression patterns of osteoarthritic (OA) and healthy cartilage. (**C**) Venn diagram depicting differentially expressed lncRNAs in osteoarthritic (OA) cartilage that are associated with OA pathogenesis.

To investigate the lncRNA gene expression landscape in OA cartilage, we re-analyzed the collected RNA-seq data from the GSE114007 dataset using the DESeq2 platform (version 1.46.0). After differential gene expression analysis in OA cartilage samples, 81 lncRNAs were characterized as significantly differentially expressed (padj < 0.05 and absolute log2FC > 0.5). More precisely, 43 lncRNAs were upregulated while 38 downregulated in OA compared to normal cartilage tissues, both with significant *p*-values (Table S1). Figure 1A,B present the volcano plot and heatmap displaying the expression profile of differentially expressed lncRNAs in OA cartilage. LncRNA LINC00671 (log2FC = 3.33), lncRNA SHANK2-AS1 (log2FC = 3.21), lncRNA LINC00520 (log2FC = 2.98), lncRNA ABCC5-AS1 (log2FC = 2.92) and lncRNA PRSS30P (log2FC = 2.84) were the top five upregulated lncRNAs in OA cartilage. Among the most downregulated lncRNAs were lncRNA LGALS8-AS1 (log2FC = −2.49), lncRNA TOB1-AS1 (log2FC = −1.78), lncRNA LINC00313 (log2FC = −1.77), lncRNA MATN1-AS1 (log2FC = −1.75) and lncRNA SMG7-AS1 (log2FC = −1.72).

The overlap between OA-associated lncRNAs acquired via the LncRNADisease platform and the 81 differentially expressed lncRNAs identified by our differential expression analysis revealed CRNDE, DNM3OS, FOXD2-AS1, MEG3 and PART1 as known OA-related lncRNAs. Among them, lncRNA MEG3 was selected for further investigation in our study (Figure 1C), as previous studies implicate MEG3 in the regulation of chondrocyte function, cartilage homeostasis, and osteoarthritis-related pathways [29,30,31].

### 3.2. Expression of lncRNA MEG3 and miR-21-5p in Normal and OA Chondrocytes

**Figure 2:**
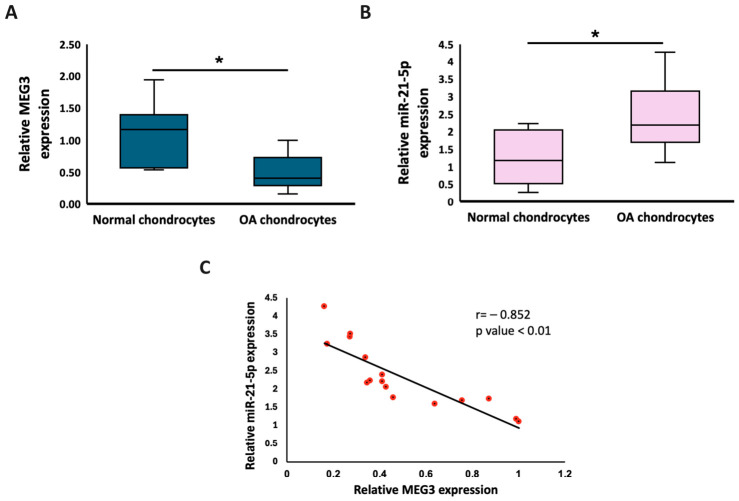
MEG3 and miR-21-5p expressions in normal and OA chondrocytes. (**A**) Relative MEG3 expression in normal (*n* = 10) and OA chondrocytes (*n* = 16), as assessed by qRT-PCR and analyzed using Mann–Whitney U test. (**B**) Relative miR-21-5p expression in normal (*n* = 10) and OA chondrocytes (*n* = 16), as assessed by qRT-PCR and analyzed using unpaired Student’s *t*-test. (**C**) Correlation between MEG3 and miR-21-5p expression in OA chondrocytes (*n* = 16) using Spearman correlation analysis. * *p* < 0.05.

Increasing evidence has shown that lncRNA MEG3 can act as a molecular sponge for miR-21-5p, binding to it and suppressing its activity and expression. Using the DIANA LncBase database, the interaction of lncRNA MEG/miR-21-5p was confirmed. To assess the potential involvement of lncRNA MEG3 and miR-21-5p in the OA phenotype of chondrocytes, lncRNA MEG3 and miR-21-5p were evaluated in cultured chondrocytes isolated from 16 OA patients and 10 healthy individuals using qRT-PCR. As shown in Figure 2A, lncRNA MEG3 was significantly decreased in OA chondrocytes compared with normal chondrocytes, consistent with the RNA-seq data, whereas miR-21-5p expression levels were upregulated in OA chondrocytes compared with chondrocytes isolated from healthy individuals (Figure 2B). Additionally, a negative correlation was found between lncRNA MEG3 and miR-21-5p expression in OA chondrocytes (Figure 2C).

### 3.3. Interaction of lncRNA MEG3 and miR-21-5p in OA Chondrocytes

**Figure 3:**
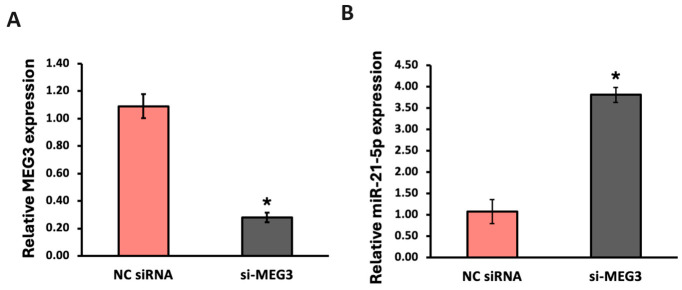
MEG3 interacts with miR-21-5p in osteoarthritis: (**A**,**B**) Bar graphs showing the expression of MEG3 and miR-21-5p in chondrocytes (*n* = 3) after treatment with 50 pmol siRNA against MEG3 for 48 h. Data were analyzed using paired Student’s *t*-test and are presented as mean ± standard error. * *p* < 0.05.

Having confirmed the reversed expression profile of MEG3 and miR-21-5p in OA chondrocytes and based on our bioinformatic analysis, we subsequently validated the interaction of lncRNA MEG3/miR-21-5p at the cellular level. Chondrocytes were treated with siRNA against lncRNA MEG3, and miR-21-5p expression levels were evaluated using RT-qPCR. Firstly, we confirmed the transfection efficiency of siRNA against lncRNA MEG3 into human chondrocytes by evaluating lncRNA MEG3 expression after transfection. LncRNA MEG3 exhibited markedly reduced expression in MEG3-siRNA-treated cells compared to NC-siRNA-treated ones (Figure 3A). Moreover, we found that lncRNA MEG3 silencing in chondrocytes markedly increased miR-21-5p expression levels compared to the NC-siRNA-treated group (Figure 3B), implying the suppressive effect of lncRNA MEG3 dysregulation on abnormal expression of miR-21-5p in OA chondrocytes.

### 3.4. Functional Enrichment Analysis and PPI Network of miR-21-5p Target Genes

**Figure 4:**
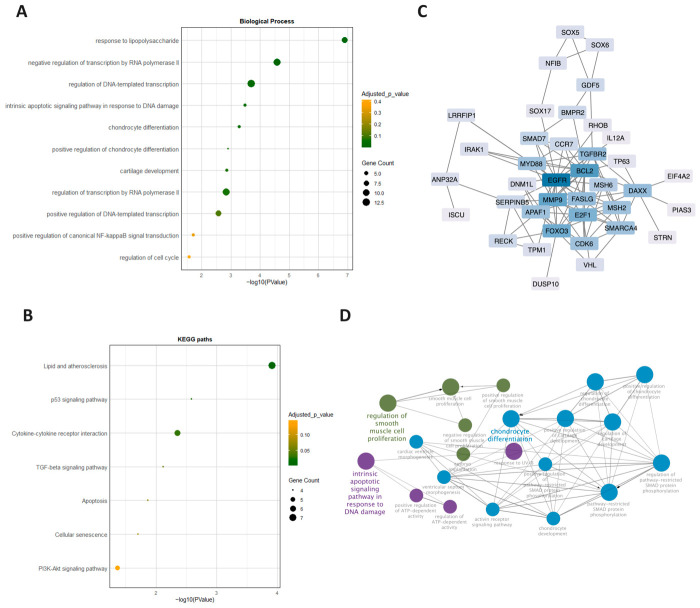
Functional enrichment and PPI network analysis of miR-21-5p target genes. (**A**) GO Biological Process analysis of miR-21-5p target genes. (**B**) KEGG terms in which miR-21-5p target genes were enriched after pathway enrichment analysis. (**C**) PPI network of miR-21-5p target genes was constructed, where nodes denote target genes and edges represent functional and physical protein associations. A minimum interaction score of 0.400 was applied as the medium-confidence threshold. (**D**) PPI network enrichment analysis using the ClueGO plugin in Cytoscape.

To examine whether miR-21-5p is implicated in processes associated with the OA phenotype of chondrocytes, experimentally validated mRNA targets of miR-21-5p were retrieved by the miRTarBase database. It was determined that miR-21-5p post-transcriptionally regulates the expression of 50 genes, among which 13 targets were reported as known OA-related genes, along with transcription factors SOX5 and TP63 (Table 3). Utilizing the DAVID database, GO Biological Process (GO-BP) analysis showed that the miR-21-5p target genes were primarily associated with response to lipopolysaccharide, regulation of gene expression, intrinsic apoptotic signaling pathway responsive to DNA damage, chondrocyte differentiation, cartilage development, regulation of canonical NF-kappaB signal transduction and the cell cycle (Figure 4A). Additionally, KEGG enrichment analysis revealed the involvement of target genes in key pathways of OA progression, such as lipid and atherosclerosis, p53 signaling pathway, cytokine–cytokine receptor interaction, cellular senescence, TGF-beta and PI3K-Akt signaling pathways (Figure 4B).

A protein–protein interaction (PPI) network was generated to delineate functional associations among miR-21-5p target genes and their involvement in OA-related pathways. The assembled network comprised 38 nodes and 104 edges (Figure 4C). Functional enrichment analysis grouped the targets into four discrete modules, notably including processes related to chondrocyte differentiation and the apoptotic signaling pathway in response to DNA damage (Figure 4D). Among the experimentally validated targets, the transcription factor SOX5—recognized for its critical role in regulating chondrocyte differentiation—was selected for further investigation.

### 3.5. MEG3 Regulates SOX5 Expression via miR-21-5p in OA Chondrocytes

**Figure 5:**
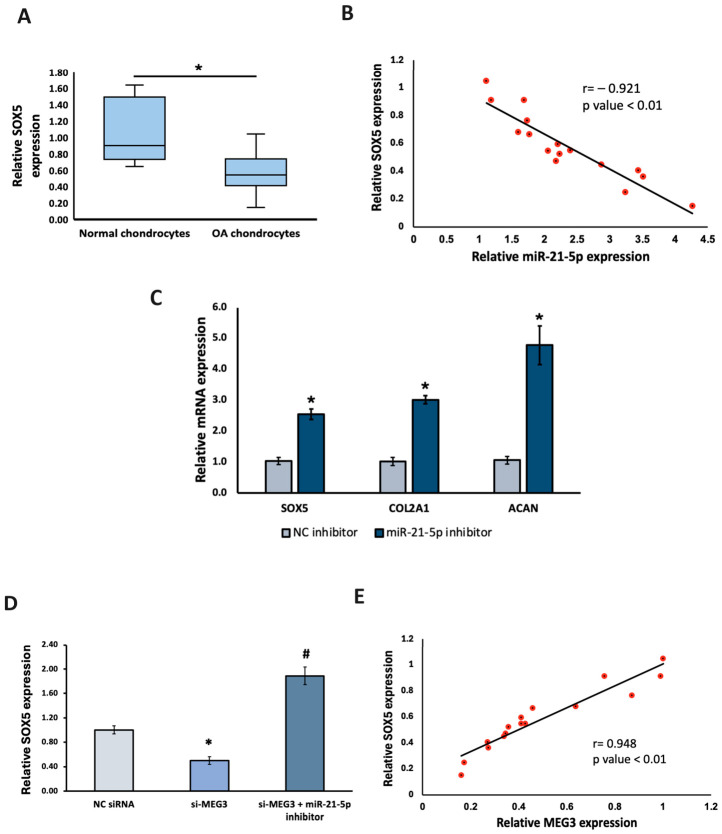
MEG3/miR-21-5p axis regulates SOX5 expression in chondrocytes. (**A**) Relative SOX5 expression in normal (*n* = 10) and OA chondrocytes (*n* = 16), as assessed by qRT-PCR and analyzed using Mann–Whitney U test. * *p* < 0.05. (**B**) Correlation between miR-21-5p and SOX5 expression in OA chondrocytes using Spearman correlation analysis. (**C**) Bar graphs showing the expressions of SOX5, COL2A1 and ACAN in chondrocytes (*n* = 3) after treatment with 25 pmol miR-21-5p inhibitor for 48 h. Data were analyzed using paired Student’s *t*-test and are presented as mean ± standard error. * *p* < 0.05. (**D**) Bar graphs showing the expression of SOX5 in chondrocytes (*n* = 3) after treatment with 50 pmol siRNA against MEG3 with or without 25 pmol miR-21-5p inhibitor. Data were analyzed using paired Student’s *t*-test and are presented as mean ± standard error. * *p* < 0.05 si-MEG3 vs NC siRNA. ^#^ *p* < 0.05 si-MEG3 + miR-21-5p inhibitor vs si-MEG3. (**E**) Correlation between MEG3 and SOX5 expression in OA chondrocytes using Spearman correlation analysis.

We first assessed SOX5 expression in OA and normal chondrocytes. OA chondrocytes exhibited significantly reduced SOX5 mRNA levels relative to normal controls (Figure 5A), and SOX5 expression was inversely correlated with miR-21-5p abundance in OA chondrocytes (Figure 5B). To investigate the possible involvement of miR-21-5p in the regulation of SOX5 expression, OA chondrocytes were transfected with a miR-21-5p inhibitor, and SOX5 mRNA was quantified by qRT-PCR. Inhibition of miR-21-5p resulted in significant upregulation of SOX5 mRNA compared with negative control (NC-treated cells). Concurrently, miR-21-5p inhibitor-treated OA chondrocytes exhibiting elevated SOX5 mRNA expression showed markedly increased levels of anabolic markers COL2A1 and ACAN relative to NC-treated cells (Figure 5C), indicating a potential regulatory relationship between miR-21-5p and SOX5 that may contribute to modulation of the chondrocyte anabolic phenotype.

Next, we examined whether miR-21-5p upregulation secondary to lncRNA MEG3 depletion affects SOX5 expression. Chondrocytes were transfected with siRNA targeting MEG3, and silencing produced pronounced decrease in SOX5 mRNA compared with NC-siRNA-treated cells; this decrease was reversed by co-treatment with miR-21-5p inhibitor (Figure 5D). Furthermore, MEG3 and SOX5 expression levels were positively correlated in OA chondrocytes (Figure 5E). Collectively, these data indicate that dysregulation of the MEG3/miR-21-5p axis contributes to aberrant SOX5 expression in OA chondrocytes and may promote OA pathogenesis via miR-21-5p-mediated suppression of SOX5.

## 4. Discussion

Abnormal expression of genes regulating the equilibrium between anabolic and catabolic processes in cartilage tissue is a key driver of osteoarthritis (OA) progression [8,9,10,32]. High-throughput sequencing technologies have identified numerous long non-coding RNAs (lncRNAs) that are differentially expressed in OA articular cartilage [13,15,16]. These lncRNAs are implicated in multiple OA-related processes, spanning chondrocyte proliferation, differentiation and apoptosis, together with inflammatory signaling, extracellular matrix metabolism, angiogenesis, and autophagy [13,15,16]. However, the precise molecular mechanisms by which lncRNAs contribute to OA pathogenesis remain largely unclear.

To characterize the lncRNA transcriptomic profile in OA cartilage, publicly available RNA-seq datasets were re-analyzed. Differential expression analysis identified 81 lncRNAs that were significantly dysregulated in OA cartilage, five of which have been previously characterized as OA related. CRNDE and DNM3OS exhibit protective effects in OA progression, as CRNDE overexpression attenuates cartilage damage and synovial inflammation in vivo [33], whereas restoration of DNM3OS expression facilitates cell proliferation and inhibits apoptosis of chondrocytes [34]. In contrast, FOXD2-AS1 appears to promote inflammation and ECM degradation via miRNA-mediated pathways, including miR-27a-3p/TLR4 and miR-206/CCND1 [35,36]. The role of PART1 remains controversial, as conflicting studies report both upregulation and downregulation in OA [30]. However, it is implicated in ECM metabolism and chondrocyte survival through modulating OA-related signaling pathways including TGFBR2/SMAD3 [37,38], which underscores the complex regulatory network of lncRNAs in OA pathogenesis.

Moreover, analysis of transcriptome high-throughput sequencing data highlighted that expression levels of lncRNA MEG3 were significantly downregulated in human OA cartilaginous tissue as well as in OA chondrocytes as confirmed by qRT-PCR. This observation aligns with prior studies reporting that decreased expression of MEG3 in OA cartilage and in in vitro OA models associated with chondrocyte homeostasis disruption, promoting apoptosis, angiogenesis and a catabolic phenotype of chondrocytes [29,30,31]. MEG3 has been shown to participate in biological processes across multiple cell types and function as a ceRNA, modulating miRNA activity such as miR-21-5p [39]. MEG3 expression was found to be inversely correlated with miR-21-5p, which was upregulated in OA chondrocytes. MEG3 knockdown induced miR-21-5p expression, supporting a regulatory role for MEG3 in controlling miR-21-5p expression in OA cartilage. It is well known that miR-21-5p is implicated in OA-related processes [40] and exhibits abnormal expression in OA tissues as well as extracellular vesicles derived from OA tissues [41,42,43], but its regulation by lncRNAs in OA has not been studied. Our study provides evidence that miR-21-5p upregulation in OA chondrocytes is mediated by MEG3 acting as a competitive endogenous RNA (ceRNA).

Interactions between lncRNAs and miRNAs are thought to play a regulatory role in cartilage-related biological processes that are disrupted in OA by modulating the expression of OA-related genes [11,12,13,14,15,16]. Enrichment analysis of miR-21-5p target genes revealed that experimentally validated miR-21-5p target genes were mainly involved in biological processes related to cartilage development and chondrocytes differentiation as well as in pathways that are dysregulated during OA including p53 signaling pathway, cytokine-cytokine receptor interaction, TGF-beta and PI3K-Akt signaling pathways. The *SOX5* gene was predicted as a target of miR-21-5p, a key transcription factor implicated in chondrocytes differentiation [44]. SOX5 expression was found to be reduced in OA chondrocytes, and its levels were inversely correlated with miR-21-5p. MiR-21-5p inhibition led to increased SOX5 expression levels in OA chondrocytes, highlighting SOX5 as a putative downstream target of miR-21-5p implicated in OA pathogenesis. Our results are consistent with previous studies unveiling the regulation of SOX5 by miR-21-5p in different animal and human tissues [45,46,47]. SOX5 enhances SOX9 transcriptional activity and drives the expression of cartilage-specific extracellular matrix genes, including COL2A1 and ACAN, thereby playing a pivotal role in cartilage homeostasis [48]. In our study, we found that miR-21-5p downregulation induced the expression of anabolic markers COL2A1 and ACAN in OA chondrocytes, suggesting that miR-21-5p acts as a suppressor of SOX5 expression in chondrocytes linked to significant consequences for cartilage integrity.

We finally proceeded to assess whether the decreased SOX5 expression in OA chondrocytes correlates to miR-21-5p upregulation mediated by MEG3, performing transfection experiments. MiR-21-5p inhibition reversed the suppressive effect of MEG3 silencing on SOX5 expression and found a negative association between miR-21-5p and SOX5 expression in OA, unveiling a novel functional regulatory system implicated in osteoarthritis onset and progression. Notably, similar regulatory patterns have been reported in other studies, where non-coding RNAs such as circ_0003800, circSEC24A, and NEAT1 act as molecular ‘’sponges’’ for miR-197-3p, miR-142-5p, and miR-193a-3p, respectively, leading to increased SOX5 expression and affecting inflammation, apoptosis and ECM degradation in OA [49,50,51]. Collectively, these findings suggest that SOX5 expression is regulated through multiple lncRNA–miRNA interactions in osteoarthritis. The proposed MEG3/miR-21-5p/SOX5 regulatory axis offers valuable insights into the molecular mechanisms involved in OA progression and may represent a candidate therapeutic axis, although further mechanistic and in vivo validation is required.

Modulating the MEG3/miR-21-5p axis may impact several other key processes implicated in osteoarthritis pathogenesis. Notably, miR-21—an extracellular miRNA—functions as a cell-to-cell communication mediator in joint tissues. Hoshikawa et al. demonstrated that synovial tissue-derived miR-21 mediates knee OA pain via TLR7 activation in a surgical rat model for OA, providing a novel strategy for pain therapy in OA patients [42]. More recently, we identified that miR-21-5p was abundant in extracellular vesicles derived from synovial tissue modulating central inflammatory markers IL-1β and IL-6, and TNF-α expression in OA chondrocytes via targeting the KLF6 transcriptional regulator [43]. Regarding MEG3 function, it has been demonstrated to regulate cell proliferation and apoptosis in OA [30], whereas Ma et al. suggested that MEG3 may also play a key part in synovial inflammation and systemic immune modulation [52], suggesting a broader role of MEG3 in processes that contribute to osteoarthritis development.

Targeting non-coding RNA networks has emerged as a promising therapeutic strategy for osteoarthritis; however, several barriers to the bench-to-bedside translation of ncRNA-based therapies still need to be overcome [53]. Developing efficient and targeted delivery systems for cartilage tissue remains a major challenge in ncRNA-based therapeutics due to the tissue’s avascular nature and dense extracellular matrix, which severely restrict molecular penetration [53]. Minimizing the off-target effects of ncRNA-based therapies also requires a deeper understanding of ncRNA biology, while issues related to RNA stability, immune activation, and long-term safety must be carefully addressed [53]. Nevertheless, emerging advances in gene delivery platforms, including lipid nanoparticles (LNPs) [54,55], exosomes [56] and hydrogels [57], offer promising solutions to overcome the barriers limiting the clinical translation of ncRNA-based interventions for cartilage regeneration.

Despite the notable aspects of our study, several limitations warrant acknowledgment. Primarily, our experimental findings are based on in vitro models, which may not recapitulate the complex microenvironment of the joint adequately; therefore, in vivo validation using animal models or human cartilage explants are crucial to verify these results. Additionally, future studies incorporating luciferase reporter, RNA pull-down, RIP, and SOX5 3′UTR validation assays will be necessary to definitively confirm the proposed regulatory interactions. Additional validation experiments, including Western blotting, immunofluorescence, and functional assays for extracellular matrix deposition and proteoglycan synthesis, would strengthen the study by confirming the effects of the MEG3/miR-21-5p/SOX5 axis at the protein level and on cartilage matrix production. Finally, since osteoarthritis is a heterogeneous disease with multiple contributing factors, the effects of disrupting the MEG3/miR-21-5p axis across different OA subtypes remain to be elucidated.

In conclusion, by integrating bioinformatic analyses with experimental validation, we unveiled that disruption of the MEG3/miR-21-5p axis was associated with reduced SOX5 expression in osteoarthritic chondrocytes. Modulation of MEG3 or miR-21-5p levels restored SOX5 expression and promoted the anabolic chondrocyte phenotype, highlighting a novel ncRNA–gene network involved in knee OA pathogenesis. Although the MEG3/miR-21-5p/SOX5 axis represents a candidate therapeutic axis for knee osteoarthritis, further studies are required to establish its therapeutic feasibility, safety, efficacy, and clinical applicability before translation into disease-modifying therapeutic strategies.

## Figures and Tables

**Table 1:** Demographic data of healthy individuals and OA patients.

Parameter	Healthy Individuals (*n* = 10)	OA Patients (*n* = 16)	*p*-Value
Age	59.20 ± 6.03	63.44 ± 6.33	0.102
Gender (%)			0.946
Female	7 (70%)	11 (68.75%)	
Male	3 (30%)	5 (31.25%)	
BMI	27.11 ± 2.07	28.86 ± 3.67	0.165
KL score	-	3.38 ± 0.16	

**Table 2:** Sequence of primers used in qRT-PCR.

Gene	Primer (5′ → 3′)	
MEG3	F	GGGGCTTCTGGAATGAGCATG
	R	TCTATGCCAGATCCTGCCTG
SOX5	F	TGCTCCAGCAACAGATCCAG
	R	ATAGCTGAAGCCTGGAGGGA
COL2A1	F	ATGACAATCTGGCTCCCAACACTGC
	R	GACCGGCCCTATGTCCACACCGAAT
ACAN	F	TGAGGAGGGCTGGAACAAGTACC
	R	GGAGGTGGTAATTGCAGGGAACA
miR-21-5p	F	GCGGCAACACCAGTCGATG
	R	TGCGTGTCGTGGAGTC
U6	F	GCTTCGGCAGCACATATACTAAAAT
	R	CTCACACCGTGTCGTTCCA
GAPDH	F	GAGTCAACGGATTTGGTCGT
	R	GACAAGCTTCCCGTTCTCAG
miR-21-5p	RT	GTCGTATCCAGTGCGTGTCGTGGAGTCGGCAATTGCACT
U6	RT	CACGGAAGCCCTCACACCGTGTCGTTC

**Table 3:** Experimentally validated mRNA targets of miR-21-5p.

***APAF1***	***SMARCA4***	***CDK6***	***VHL***	***EIF4A2***	***TP63***	***IRAK1***	***DUSP10***	***MYD88***	***SOX17***
***FASLG***	***SOX5***	***CCR7***	***CDK2AP1***	*FOXO3*	*LRRFIP1*	*SMAD7*	*ISCU*	*NFIB*	*WWC2*
***RHOB***	*STRN*	*DAXX*	*ANP32A*	*MSH6*	*DNM1L*	***MMP9***	***DDAH1***	*PFKFB2*	*SLC16A10*
***BCL2***	***TGFBR2***	*E2F1*	***GDF5***	*HPGD*	*RASGRP1*	*MSH2*	*CDIP1*	*SERPINB5*	*ANKRD46*
***BMPR2***	*TPM1*	*EGFR*	*RECK*	*IL12A*	*PIAS3*	*MTAP*	*SOX6*	*SERPINI1*	*CASC2*

Target genes associated with osteoarthritis are highlighted in bold.

## Data Availability

Data are contained within the article and Supplementary Materials. Data are available upon request to the corresponding author.

## Supplementary Materials

The following supporting information can be downloaded at: https://www.mdpi.com/article/10.3390/genes17070748/s1, Table S1: Significant DE lncRNAs OA vs. healthy cartilage (adjusted *p* value < 0.05; absolute Log2FoldChange > 0.5), as calculated by DESeq2.
